# Supplementary material for: Gene-based association analysis identifies 190 genes affecting neuroticism
Source: Sci Rep. 2021 Jan 28;11:2484. doi: 10.1038/s41598-021-82123-5 (PMC7844228; doi:10.1038/s41598-021-82123-5)
Supplement: Supplementary file 1 — Supplementary Information 1. [file 41598_2021_82123_MOESM1_ESM.zip › Supplementary Methods.pdf]

Gene-based association analysis identifies 190 genes affecting neuroticism

Nadezhda M. Belonogova[1], Irina V. Zorkoltseva[1], Yakov A. Tsepilov[1,2], Tatiana I. Axenovich[1,2,*]

[1] Institute of Cytology and Genetics, Siberian Branch of the Russian Academy of Sciences, Novosibirsk, Russia

[2] Department of Natural Sciences, Novosibirsk State University, Novosibirsk, Russia

[*] Corresponding author:

Tatiana I. Axenovich

Tel.: +7 383 363 4980

Fax: +7 383 333 1278

e-mail: tatiana.aksenovich@gmail.com

Supplementary methods

Methods for gene-based association analysis

Gene-based association analysis is the popular approach for gene mapping. The increased power of this approach is ensured by the simultaneous analysis of all variants within gene (Eichler et al. 2010; Li and Leal 2008). Many methods of gene-based analysis use different types of multiple linear regression models. Recently, it was demonstrated that all methods of gene-based association analysis based on the linear regression models can use GWAS summary statistics instead of individual genotypes and phenotypes (Svishcheva 2019). Large number of summary statistics is now available in open databases as well as the packages for their analysis. In particular, we recently developed sumFREGAT package (Svishcheva et al. 2019) for gene-based association analysis using summary statistics with the help of following methods: burden (BT), SKAT and SKAT-O tests, analyses of multiple linear regression (MLR), principal components (PCA), functional data analysis (FLR), aggregated Cauchy association test (ACAT), and others.

For each SNP, a z-score test compares two hypotheses: H_0: $\beta = 0$ against H_1: $\beta \neq 0$, where β is traditionally estimated using the maximum likelihood approach. A vector of z-scores, *Z*, obtained one by one for each SNP in the gene is defined as

$$Z = \frac{V^{-1}\tilde{G}^T\tilde{y}}{\sqrt{n}\sigma_y}. \qquad (2)$$

Here $\tilde{y}$ denotes the centered values of phenotypes y for n persons, $\tilde{G}$ is the centered ($n\times m$) matrix genotypes G for m variants and V is an ($m\times m$) diagonal matrix of the square roots of genotypic variances. Under the null hypothesis, Z follows the multivariate normal distribution $N(0,U)$, where U is calculated as an ($m\times m$) matrix of correlations between the genotypes of these variants (Conneely and Boehnke 2007):

$$U = \frac{V^{-1}\tilde{G}^T\tilde{G}V^{-1}}{n}$$

SKAT-O method

We demonstrated that gene-level tests for BT, SKAT and SKAT-O methods can be described as (Svishcheva et al. 2019):

$$Q_{BT} = n(Z^TVWe)^2,$$

$$Q_{SKAT} = nZ^TVWWVZ,$$

$$Q_{SKAT\text{-}O} = (1-\rho)\,Q_{SKAT} + \rho\,Q_{BT},$$

where e is a ($m\times 1$) vector of units, W is a diagonal matrix of weights that are defined by the user to control the impact of each genetic variant, and ρ can be interpreted as a pair-wise correlation among the genetic effects β (Lee et al. 2013).

Under the null hypothesis, Q_{BT} follows a scaled χ^2 distribution with one degree of freedom, $\lambda\chi_1^2$, where $\lambda = (e^T WVUVWe)n$, and Q_{SKAT} follows a weighted sum of χ^2 distributions with one degree of freedom, $\sum \lambda\chi_1^2$, where λ is an $(m\times1)$ vector of the eigenvalues of the kernel matrix, i.e. $\lambda = eig(WVUVW)n$.

PCA method

PCA method serve to reduce the number of the predictors in the regression model by generating an orthogonal basis set of $k < m$ elements. For PCA, these elements are the first k principal components containing a large proportion of information about the genotype data.

For PCA method, the F-test is used to test for the null hypothesis H_0: $\beta = 0$ against H_1: $\beta \neq 0$:

$$F = \frac{R^2(n-k-1)}{(1-R^2)k},$$

where

$$R^2 = \frac{Z^T VWC(C^T WVUVWC)^{-1} C^T WVZ}{n}.$$

Here C is given as a truncated matrix of right singular vectors obtained from the singular value decomposition of the weighted genotype matrix. Truncating is achieved by considering only the first k largest squared singular values that account for 80-90% of the total genotype variance observed in the genomic region.

The p-value for the gene-level test is defined by an F-distribution with k and n-k-1 as the numbers of degrees of freedom.

ACAT method

The recently proposed ACAT test uses a combination of transformed variant p-values (Liu et al. 2019). For combination of p-values obtained for each within-gene variant, ACAT-V test statistic is given by:

$$T_{ACAT-V} = \frac{1}{\sum w_j^2} \sum_{j=1}^{m} w_j^2 \tan((0.5 - p_j)\pi)$$

where m is the number of variants, p_j is the association p-value and w_j is the weight of the j-th variant.

Similarly, an omnibus test to aggregate information from three different methods was defined as:

$$T_{ACAT-O} = 1/3[\tan\big((0.5 - p_{SKAT-O})\pi\big) + \tan\big((0.5 - p_{PCA})\pi\big) + \tan\big((0.5 - p_{ACAT-V})\pi\big)].$$

Both ACAT tests are well approximated using a Cauchy distribution under the null hypothesis and its p-value can be obtained analytically as:

$$p_{ACAT} \approx \frac{1}{2} - \frac{\arctan(T_{ACAT})}{\pi}.$$

Parameters used in the current study

For all methods, weights were defined by MAF using Beta distribution: beta.par = c(1, 1) in all tests. Other options were set to the default values, including var.fraction = 0.85 for PCA.

References

Conneely KN, Boehnke M (2007) So many correlated tests, so little time! Rapid adjustment of P values for multiple correlated tests Am J Hum Genet 81:1158-1168 doi:10.1086/522036

Eichler EE, Flint J, Gibson G, Kong A, Leal SM, Moore JH, Nadeau JH (2010) Missing heritability and strategies for finding the underlying causes of complex disease Nat Rev Genet 11:446-450 doi:10.1038/nrg2809

Lee S, Teslovich TM, Boehnke M, Lin X (2013) General framework for meta-analysis of rare variants in sequencing association studies Am J Hum Genet 93:42-53 doi:10.1016/j.ajhg.2013.05.010

Li B, Leal SM (2008) Methods for detecting associations with rare variants for common diseases: application to analysis of sequence data Am J Hum Genet 83:311-321 doi:10.1016/j.ajhg.2008.06.024

Liu Y, Chen S, Li Z, Morrison AC, Boerwinkle E, Lin X (2019) ACAT: A Fast and Powerful p Value Combination Method for Rare-Variant Analysis in Sequencing Studies Am J Hum Genet 104:410-421 doi:10.1016/j.ajhg.2019.01.002

Svishcheva GR (2019) A generalized model for combining dependent SNP-level summary statistics and its extensions to statistics of other levels Sci Rep 9:5461 doi:10.1038/s41598-019-41827-5

Svishcheva GR, Belonogova NM, Zorkoltseva IV, Kirichenko AV, Axenovich TI (2019) Gene-based association tests using GWAS summary statistics Bioinformatics 35:3701-3708 doi:10.1093/bioinformatics/btz172

Polygene pruning

The purpose of this procedure was to weaken the influence of association signals located outside the analyzed region by excluding SNPs that are in high LD with more significant SNPs outside the region.

The procedure is as follows:

1. For each SNP with a p-value < 0.0001 (index SNP), we formed a list of linked SNPs (clump) from all other SNPs that are in high LD (estimated with 1000G, $r^2 > 0.5$), located within 5 000 kb from the index SNP and having 0.05 > p-value > p-value of the index SNP.

   For clumping, we used PLINK 1.9 with the following options:

   --clump-p1 0.0001

--clump-p2 0.05

--clump-r2 0.5

--clump-kb 5 000

--clump-range-border 5 000

--clump-allow-overlap

--clump-best

With the --clump-best option, PLINK does not use greedy algorithms and considers all SNPs when constructing each clump. This allowed us to obtain the most complete clumps for all index SNPs. However, some of such clumps contained SNPs with a lower p-value than that of its index SNP. An additional step was to filter these SNPs out of the clumps.

2. For each gene, we formed a list of index SNPs located within or close to the gene (within 5 000 kb from its border). Then we subdivided the list of index SNPs into two sets: one for those located inside and one for those located outside the analyzed region (defined according to one of three types of analysis described in 'Regions of interest' section).
3. For each analyzed region, we removed SNPs if they are in the clumps of index SNPs outside the analyzed region.
4. We re-analyzed the region using the SNPs that remained.

SMR/HEIDI analysis

SMR/HEIDI analysis was conducted as described by Zhu et al. (2016).

HEIDI statistics was calculated as $T_{HEIDI} = \sum_i^m z_{d(i)}^2$, where m is the number of SNPs selected for analysis, $z_{d(i)} = {}^{d_i}/_{SE_{(d_i)}}$ and $d_i = \beta_{SMR_i} - \beta_{SMR\ (lead\ SNP)}$.

SNP selection was performed as follows:

1) We defined a set of eligible markers within ±250 kb from the lead SNP in the primary GWAS, which had $\chi^2 > 10$ in the primary GWAS, and for which the results were reported in the secondary GWAS;
2) Created an empty "target" and an empy "rejected" SNP set;
3) From the primary GWAS, selected the SNP with the lowest p-value;
4) If this SNP had $r^2 > 0.9$ with any SNP in the target SNP set, we added it to the "rejected" set. The LD matrix (r^2) was computed with PLINK 1.9 (https://www.cog-genomics.org/plink2) using 1 000 Genomes data for 503 European individuals (http://www.internationalgenome.org/data/);
5) Otherwise, it was added to the "target" set;

6) The procedure was repeated from step 3 until either eligible SNP set was exhausted or the “target” set had 20 SNPs. If we could not select at least three SNPs, no test was performed.

Analysis was conducted using Python 3.5 as the main programming language.

We selected the genes that were significant in gene-based analysis after pruning using non-coding intronic variants only (164 genes in total). Further, we filtered genes if a p-value for the most associated SNP within the gene (top SNP) was more than 5×10^{-8}. It should be noted that the SMR/HEIDI procedure searches for overlapping between both GWAS in analysis. The top SNP for neuroticism was not always present in the expression GWAS panel; in that case, we searched for the second SNP most associated with neuroticism that was also present in the expression GWAS panel (Proxy top SNP). If there was no Proxy top SNP with $r^2 \geq 0.8$ with the top SNP, we removed this locus from analysis. We considered a locus pleiotropic with expression level if p-values of association for the Proxy top SNP in both neuroticism and expression level GWAS were significant genome-wide (p-value $< 5\times10^{-8}$). The significance threshold for HEIDI tests was set at a p-value = 0.001 ($p < 0.001$ corresponds to the rejection of the pleiotropy hypothesis).

Zhu Z, Zhang F, Hu H, Bakshi A, Robinson MR, Powell JE, Montgomery GW, Goddard ME, Wray NR, Visscher PM et al. (2016) Integration of summary data from GWAS and eQTL studies predicts complex trait gene targets Nat Genet 48:481-487 doi:10.1038/ng.3538
